# Impact of opportunistic testing in a systematic cervical cancer screening program: a nationwide registry study

**DOI:** 10.1186/s12889-015-2039-0

**Published:** 2015-07-21

**Authors:** Mette Tranberg, Mette Bach Larsen, Ellen M. Mikkelsen, Hans Svanholm, Berit Andersen

**Affiliations:** Department of Public Health Programs, Randers Regional Hospital, Skovlyvej 1, DK-8930 Randers, NØ Denmark; Department of Clinical Epidemiology, Aarhus University Hospital, Olof Palmes Allé 43-45, DK-8200 Aarhus N, Denmark; Department of Pathology, Randers Regional Hospital, Skovlyvej 1, DK-8930 Randers, NØ Denmark

**Keywords:** Mass screening, Opportunistic testing, Vaginal smears, Sociodemographic characteristics, Uterine cervical dysplasia, Uterine cervical neoplasm, Primary prevention

## Abstract

**Background:**

Systematic screening for precancerous cervical lesions has resulted in decreased incidence and mortality of cervical cancer. However, even in systematic screening programs, many women are still tested opportunistically. This study aimed to determine the spread of opportunistic testing in a systematic cervical cancer screening program, the impact of opportunistic testing in terms of detecting cytological abnormalities and examine the associations between sociodemography and opportunistic testing.

**Methods:**

A nationwide registry study was undertaken including women aged 23–49 years (n = 807,624) with a cervical cytology between 2010 and 2013. The women were categorised into: 1) screening after invitation; 2) routine opportunistic testing, if they were either tested more than 9 months after the latest invitation or between 2.5 years and 3 years after the latest cervical cytology and 3) sporadic opportunistic testing, if they were tested less than 2.5 years after the latest cervical cytology. Cytological diagnoses of women in each of the categories were identified and prevalence proportion differences (PPD) and 95 % confidence intervals (CIs) were used to explore group differences. Associations between sociodemography and undergoing opportunistic testing were established by multinomial logistic regression.

**Results:**

In total, 28.8 % of the cervical cytologies were due to either routine (20.7 %) or sporadic (8.1 %) opportunistic testing. Among women undergoing routine opportunistic testing, a larger proportion had high-grade squamous intraepithelial abnormalities than invited women (PPD: 0.6 %, 95 % CI: 0.03–1.17 %). A similar proportion of cytological abnormalities among women undergoing sporadic opportunistic testing and invited women was found. In multivariate analyses, younger age, being single or a social welfare recipient and residence region (North Denmark) were especially associated with opportunistic testing (routine or sporadic).

**Conclusions:**

One fourth of cervical cytologies in this study were collected opportunistically. Compared to invited women, women undergoing routine opportunistic testing were more likely to be diagnosed with abnormal cytologies. Hence, routine opportunistic testing might serve as an important supplement to the systematic screening program by covering non-participating women who may otherwise be tested with a delay or not tested at all. Among women tested more often than recommended (sporadic testing), no benefits in terms of detecting more cytological abnormalities were identified.

## Background

A nationally organised cervical cancer screening program is an important intervention for preventing cervical cancer. Such programs target detection of precancerous cervical lesions so that women can be preventively treated and avoid developing cancer, and their use has been followed by a decrease in incidence and mortality rates of cervical cancer in many European countries [1–4].

In systematic cervical cancer screening programs, healthy women are continuously invited for screening at regular time intervals [5–7]. Even in countries with organised programs, however, many women are tested opportunistically and have a cervical cytology taken outside the organised program schedule, usually initiated by the woman herself or by her general practitioner (GP) [8–12]. For example, the British National Health Service cervical cancer screening program estimated the proportions of opportunistic testing to be 17 % and 43 % in 3- and 5-year screening policy areas, respectively, resulting in a considerably reduced average screening interval for women, especially in 5-year policy areas [8].

Some authors have argued that opportunistic testing is not cost effective and should not be performed [9, 13, 14], but the significance of opportunistic testing in detecting precancerous cervical lesions remains unclear. Furthermore, many countries suffer from low participation rates in their systematic screening programs [15], which reduces the effect of the programs. In particular, women who are underserved or of lower socioeconomic status tend not to participate in cervical cancer screening programs [16], which further reduces the effect of the programs because this population also is at higher risk of developing cervical cancer [17].

In this register-based nationwide study, we therefore aimed to determine the proportion of cytology specimens taken opportunistically in a cervical cancer screening program and to evaluate the significance of opportunistic testing in terms of detecting cytological abnormalities. Furthermore, we examined the association between sociodemographic factors and undergoing opportunistic testing.

## Methods

This study was designed as a nationwide cross-sectional study in Denmark based on registry data. Denmark has a total of 5.6 million inhabitants, with 1.5 million women in the target population for cervical cancer screening [18]. Systematic cervical cancer screening was introduced in the 1960s in some counties and non-systematically implemented in the rest of the country until nationwide coverage was achieved in 2007 [19].

The policy of cervical cancer screening is defined nationally and administered by Denmark’s five regions. Every third year, women ages 23 to 49 years are invited for cervical cancer screening while women ages 50 to 64 years are invited every fifth year. A woman in the target population receives a personal invitation 3 or 5 years after her last cervical cytology unless she has declined to be a part of the program. The invitation advises the woman to book an appointment at her GP for a pelvic examination. When the cytology has been taken by the GP it will be mailed to the local department of pathology for analysis. If a cervical cytology is not obtained, a reminder is sent out 3 months (90 days) after the primary invitation and potentially a second reminder after 6 months (180 days) [18]. If no cytology is taken within 3 or 5 years after the last invitation, a new invitation is mailed. Danish guidelines do not recommend cervical cancer screening during pregnancy, but testing can be resumed 8–12 weeks postpartum [20]. Immigrants receive an invitation for cervical cancer screening when they obtain a Danish civil registration number (CRN). Outside the organised screening program, a GP or a gynaecologist can obtain an opportunistic cervical cytology at any time. The local pathology department analyses opportunistic as well as invitational cervical cytologies.

If a woman has an abnormal or inadequate cervical cytology result, she will be enrolled in a surveillance program [20]. In the surveillance program, women are tested more frequently than recommended in the screening program and therefore receive no invitation for screening. Duration and intensity of the surveillance program depend on the severity of the identified abnormality. In the Danish program, both invitational and opportunistic cervical cytologies are performed free of charge [20].

### Study population

**Fig. 1:**
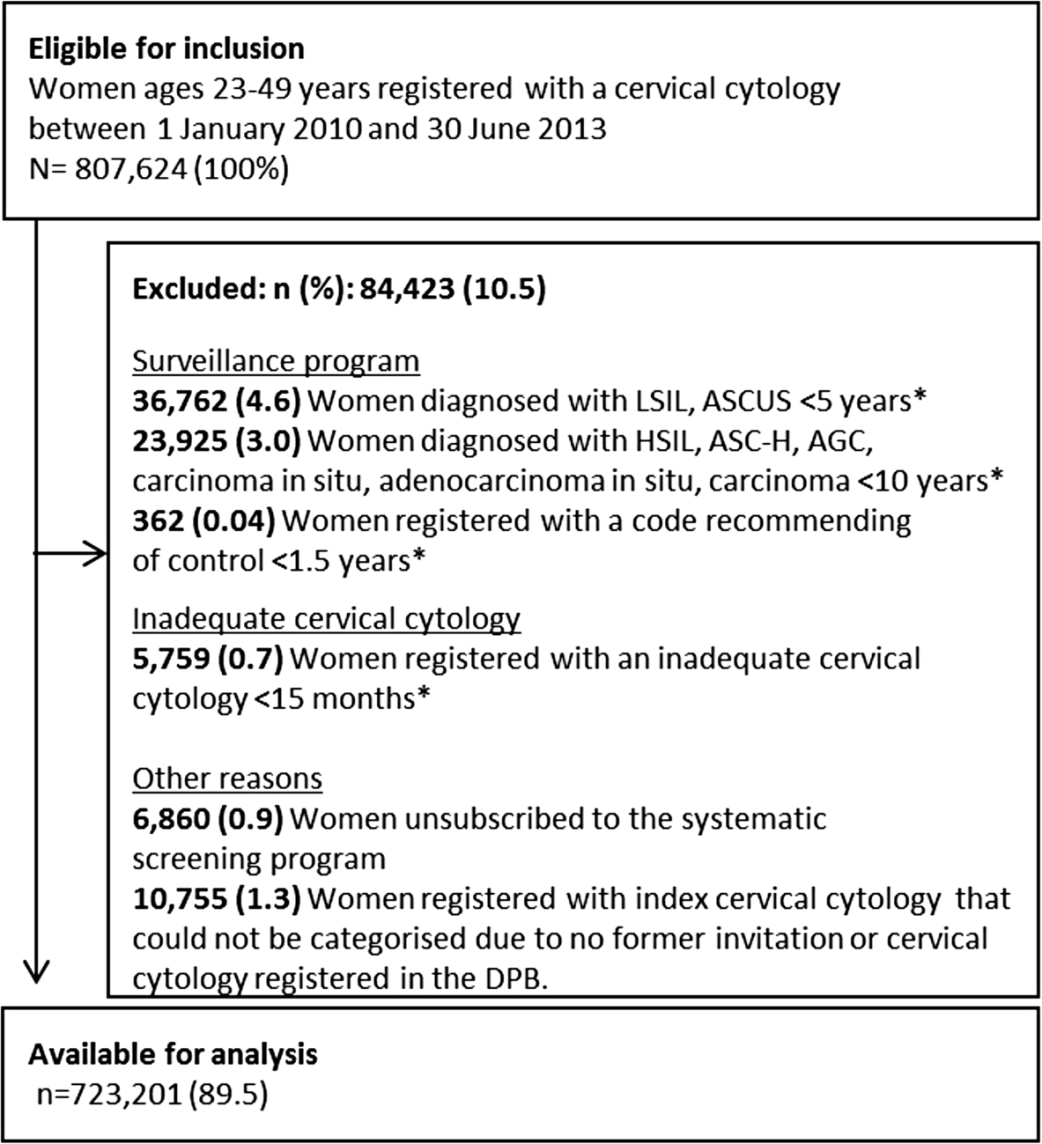
Flowchart for the study population ASCUS: atypical Squamous Cells of Undetermined Significance. ASC-H: atypical Squamous Cells cannot exclude HSIL, AGC: atypical Glandular Cells. LSIL: low grade Squamous Intraepithelial Lesion. HSIL: high-grade Squamous Intraepithelial Lesion. *) Prior to the index cytology

Our study population included women ages 23–49 years who were registered in the Danish National Pathology Data Bank (DPB) with a cervical cytology between 1 January 2010 and 30 June 2013 (3.5 years). Women aged 50–64 years were not included in this study because of a different screening interval as compared to women aged 23–49 years. Study inclusion was based on a first cervical cytology registered after 1 January 2010 (the index cervical cytology). Exclusion criteria were as follows (Fig. 1): abnormal cervical cytology finding prior to the index cervical cytology, indicating that the index cervical cytology could be part of a surveillance program (see Appendix 1 for used codes); an inadequate cervical cytology prior to the index cervical cytology, indicating that the index cervical cytology was taken, for example, because of a lack of cytological material for diagnostic assessment; being unsubscribed to the systematic screening program and therefore not receiving any invitations; and/or being registered with an index cytology but having no former invitation or cervical cytology.

### Data sources

#### Cervical cytology

We collected data on cervical cytology from the DPB. This database use the Systematised Nomenclature of Medicine (SNOMED) to store detailed records of all pathology specimens, including cervical cytology analysed in Denmark since 1997 [21]. For cervical cytologies, the Bethesda classification has been recommended since 2010 [22]. Before 2010, the most used classification system for cervical cytologies was a modified World Health Organization classification [23], which can easily be translated into the Bethesda classification (see Appendix 1).

In DPB, we identified cervical cytologies using the SNOMED codes of cervix uteri: T8X3* [18]. For each cervical cytology, we identified the date of the last invitation, date of sample acquisition, and date of the latest cytology taken before the index cytology. Data on cytological diagnosis were retrieved using the SNOMED codes according to the Bethesda classification, as follows: normal cells; atypical squamous cells of undetermined significance (ASCUS); atypical squamous cells, cannot exclude high-grade squamous intraepithelial lesion (HSIL) (ASC-H); atypical glandular cells (AGC); low-grade squamous intraepithelial lesion (LSIL); HSIL; carcinoma in situ and adenocarcinoma in situ (AIS); carcinoma (including squamous carcinoma and adenocarcinoma); inadequate cervical cytology (not suitable for diagnosis); and others, e.g., necrosis (Appendix 1).

#### Sociodemography

Data on sociodemographic characteristics of women in the study population by the end of 2012 were obtained from The Danish Integrated Database for Labour Market Research (IDA) [24], which is annually updated for all Danish citizens. Educational level was classified according to UNESCO classification as low (≤10 years), middle (11–15 years), or higher education (>15 years). Occupation was classified as employed; self-employed and chief executive; unemployed or receiving supplementary benefits other than social welfare; retired; social welfare recipient; or other. Marital status was classified as married or living in a registered partnership, cohabitating, or single. Ethnicity was divided into Danish, immigrants from western countries, or immigrants from non-western countries, according to Statistics Denmark’s definition of developed countries [25]. Residence region was classified as North Denmark, Central Denmark, Region of Southern Denmark, Capital Region of Denmark, or Region Sealand. Each woman’s age at the date of the index cervical cytology was calculated by subtracting the woman’s date of birth from the date of the index cytology. Age was categorised corresponding to previous studies as 23–28 years, 29–34 years, 35–42 years, or 43–49 years [17, 26].

### Data handling

Every Danish citizen has a unique 10-digit CRN including the date of birth and four additional random digits [27]. Every contact with the health care system and all information on sociodemographic factors are registered through this CRN [27], which allowed us to link data on cervical cytology from DPB to data on sociodemography from the IDA database.

#### Categorisation of cervical cytology

As no registry data are available to distinguish the invitational cytologies from opportunistic cytologies, the cervical cytologies were categorised into three groups according to the time span between the date of the index cervical cytology and either the 1) date of the last invitation or 2) date of last cervical cytology, whatever was closest to the index cervical cytology. Categorisation was done as follows: A woman was categorised as being ‘screened after invitation’ if her index cervical cytology was registered within ≤270 days (9 months) after her latest invitation. The group of women who underwent opportunistic testing was divided into routine or sporadic testing. Thus, we defined a woman as undergoing ‘routine opportunistic testing’ if one of two conditions was present: the index cervical cytology was taken 9 months to 3 years (271 days to 1080 days) after the woman’s latest invitation (extended screening interval); or the index cervical cytology was performed 2.5 to 3 years (901–1080 days) after the woman’s latest cervical cytology, which is slightly before an invitation would have been sent. A woman with no invitation but an index cervical cytology taken less than 2.5 years after her latest cervical cytology was defined as having undergone ‘sporadic opportunistic testing’*.* This latter type of cervical cytology is taken at a shorter screening interval than recommended.

### Statistics

#### Cervical cytology diagnoses

Prevalence proportion differences (PPDs) and prevalence proportion ratios (PPRs) with 95 % confidence intervals (CIs) were used to explore differences in the distribution of cytological diagnoses between women undergoing routine opportunistic testing and women screened after invitation, and between women having sporadic opportunistic testing and women being screened after invitation. Further, the analyses were stratified by age: 23–28 years, 29–34 years, 35–42 years and 43–49 years.

In a sensitivity analysis, we explored the impact of giving the women a longer time frame to respond to the invitation. Thus, we used ≤365 days as a cut-off value to differentiate between women screened after invitation and women undergoing opportunistic testing, which is in accordance with the definition in the Danish quality database for cervical cancer screening [18].

#### Sociodemography

Sociodemographic characteristics were reported for each of the three cervical cytology groups using proportions and chi-square tests.

We used a multinomial logistic regression model to estimate odds ratios (ORs) with 95 % CIs for the associations between sociodemography and routine opportunistic testing or sporadic opportunistic testing. Women being screened after invitation served as the reference group. Unadjusted analyses were performed with each independent variable followed by a multivariate model adjusting for all independent variables. Independent variables included age groups, residence region, ethnicity, marital status, occupation, and education. Identification of covariates was based on a literature review [26, 28, 29]. Initially, to check for multicollinearity between independent variables, the mean variance inflation factor (VIF) was calculated. Values above 10 indicate multicollinearity [30].

All statistical analyses were conducted using STATA 13.0.

### Ethics

The study was approved by the Danish Data Protection Agency (j.no. 2007-58-0010). According to Danish Legislation and the Central Denmark Region committees on biomedical research ethics, the study did not need ethics approval because it was based solely on registry data.

## Results

### Sample characteristics

A total of 807,624 women were registered with a cervical cytology in the DPB during the study period. Of these, a total of 84,423 (10.5 %) were excluded from the study population, leaving 723,201 (89.5 %) for the analyses. The main reason for exclusion was a recent cytological diagnosis consistent with being in a surveillance program (61,049 women = 7.6 %) (Fig. 1).

In total, 514,833 women (71.2 %) were categorised as screened after invitation, 149,778 women (20.7 %) as having undergone routine opportunistic testing, and 58,590 women (8.1 %) as having had sporadic opportunistic testing. Among women undergoing routine opportunistic testing, a total of 111,971 (15.5 %) were tested more than 9 months after the latest invitation, and 37,807 women (5.2 %) were tested 2.5 to 3 years after the latest cytology, shortly before having received an invitation . Mean age was 36.7 years for invited women, 35.2 years for women undergoing routine opportunistic testing, and 36.8 years for women having sporadic opportunistic testing (data not shown). Table 1 shows the distribution of sociodemographic variables among women included in the study. Women who had routine or sporadic opportunistic testing differed significantly from invited women in all sociodemographic characteristics (chi^2^ p < 0.01).

**Table 1:** Sociodemographic characteristics of women screened after invitation and women undergoing routine or sporadic opportunistic testing

Variables	Screened after invitation^1^		Routine opportunistic testing^2^		Sporadic opportunistic testing^3^	
	n = 514,833		n = 149,778		n = 58,590	
	n	%	n	%	n	%
Age (years)						
23–28	104,488	20.3	36,357	24.3	10,417	17.8
29–34	93,912	18.2	36,896	24.6	13,004	22.2
35–42	158,989	30.9	44,098	29.4	18,280	31.2
43–49	157,444	30.6	32,427	21.7	16,889	28.8
Residential region						
North Denmark	40,778	7.9	16,649	11.1	6,658	11.4
Central Denmark	113,977	22.1	33,259	22.2	12,088	20.6
Region of Southern Denmark	109,159	21.2	25,485	17.0	9,647	16.5
Capital Region of Denmark	167,310	32.5	50,859	34.0	23,016	39.3
Region Sealand	72,537	14.1	18,076	12.1	5,174	8.8
Ethnicity						
Danish	464,711	90.3	129,550	86.5	52,621	89.8
Western immigrants	18,739	3.6	8,032	5.4	1,887	3.2
Non-western immigrants	19,910	3.9	6,585	4.4	2,011	3.4
Marital status						
Single	125,215	24.3	44,120	29.5	16,965	29.0
Married/registered partnership	260,265	50.6	65,625	43.8	27,702	47.3
Cohabitating	100,092	19.4	34,782	23.2	12,347	21.1
Occupation						
Employed	383,011	74.4	102,797	68.6	42,606	72.7
Self-employed/chief executive	23,786	4.6	6,736	4.5	3,047	5.2
Unemployed/benefits^4^	41,633	8.1	14,547	9.7	5,607	9.6
Retired	2	0.0	NA	NA	NA	NA
Social welfare recipients	15,902	3.1	8,047	5.4	2,307	3.9
Other	33,283	6.5	10,825	7.2	2,714	4.6
Education (years)						
≤10	89,436	17.4	28,750	19.2	10,278	17.5
11–15	213,607	41.5	54,818	36.6	22,267	38.0
>15	184,185	35.8	53,984	36.0	22,845	39.0

Numbers and proportion vary because of missing data.

1) Cervical cytology obtained within 270 days after latest invitation.

2) Cervical cytology obtained between 271 days to 3 years after latest invitation or 2.5 to 3 years after latest cervical cytology.

3) Cervical cytology obtained less than 2.5 years after latest cervical cytology.

4) State benefits in relation to sickness, education, leave benefits, disability pension, and student grants

### Cytological diagnosis

Table 2 presents the distribution of cytological diagnoses among women in the three categories. Women who had routine and sporadic opportunistic testing had a lower proportion of normal cytological diagnoses as compared to invited women (PPD: −1.7 %, CI: −1.87 to −1.52 %; and PPD: −1.1 %, CI: −1.34 to −0.86 %, respectively). Among women undergoing routine opportunistic testing, a higher proportion of HSIL diagnoses was seen as compared to invited women (PPD: 0.6 %, CI: 0.03–1.17 %, PPR: 1.60, CI: 1.52–1.68). In addition, women undergoing routine opportunistic testing had a 3.4-fold increased relative risk of being diagnosed with carcinoma as compared to invited women. We observed only minor differences in the prevalence of abnormal cytological diagnoses between invited women and women undergoing sporadic opportunistic testing (HSIL: PPD: −0.2 %, CI: −1.05 to 0.65 % and carcinoma: PPD: 0.01 %, CI: −0.31 to 0.29 %). The distribution of normal, HSIL, carcinoma in situ and carcinoma cytological diagnosis stratified by age group are presented in Table 3. The PPDs for HSIL and carcinomas were similar across age groups. For sporadic opportunistic testing, women aged 29–34 years had the lowest relative risk for being diagnosed with HSIL compared to women aged 43–49 years.

**Table 2:** Cytological diagnosis for women screened after invitation and women undergoing routine or sporadic opportunistic testing

	Screened after invitation^1^		Routine opportunistic testing^2^				Sporadic opportunistic testing^3^			
	n = 514,833 **(71.2 %)**		n = 149,778 **(20.7 %)**				n = **58,590 (8.1 %)**			
	n	%	n	%	PPD % (95 % CI)^4^	PPR (95 % CI)^5^	n	%	PPD % (95 % CI)^6^	PPR (95 % CI)^7^
Cytological diagnosis										
Normal cells	478,855	(93.0)	136,797	(91.3)	**−1.7 (−1.87; −1.52)**	**0.98 (0.98;0.98)**	53,866	(91.9)	**−1.1 (−1.34; −0.86)**	**0.99 (0.99;0.99)**
ASCUS	10,868	(2.1)	3,674	(2.5)	0.4 (−0.17; 0.95)	**1.16 (1.12;1.21)**	1,361	(2.3)	0.2 (−0.64;1.04)	**1.10 (1.04;1.16)**
ASC-H	2,175	(0.4)	846	(0.6)	0.2 (−0.38; 0.78)	**1.34 (1.24;1.45)**	250	(0.4)	0.0 (−0.83; 0.83)	1.01 (0.89;1.15)
AGC	634	(0.1)	236	(0.2)	0.1 (−0.52; 0.72)	**1.28 (1.10;1.49)**	99	(0.2)	0.1 (−0.81; 1.01)	**1.37 (1.11;1.70)**
LSIL	7,857	(1.5)	2,760	(1.8)	0.3 (−0.26; 0.86)	**1.21 (1.16;1.26)**	945	(1.6)	0.1 (−0.74; 0.94)	1.06 (0.99;1.13)
HSIL	4,791	(0.9)	2,228	(1.5)	**0.6 (0.03; 1.17)**	**1.60 (1.52;1.68)**	408	(0.7)	−0.2 (−1.05; 0.65)	**0.75 (0.68;0.83)**
Carcinoma in situ + AIS	106	(0.02)	45	(0.03)	0.01 (−0.56; 0.58)	1.46 (1.00;2.07)	14	(0.02)	0.0 (−0.79; 0.79)	1.16 (0.66;2.03)
Carcinoma^8^	42	(0.01)	42	(0.03)	0.02 (−0.58; 0.62)	**3.44 (2.24;5.27)**	2	(0.0)	−0.01 (−0.31; 0.29)	0.42 (0.10;1.72)
Inadequate cervical cytology^9^	9,109	(1.8)	2,988	(2.0)	0.2 (−0.37; 0.77)	**1.13 (1.08;1.17)**	1,538	(2.6)	0.8 (−0.04; 1.64)	**1.48 (1.40;1.56)**
Others^10^	29	(0.01)	8	(0.01)	0.0 (−0.78; 0.78)	0.95 (0.43;2.07)	1	(0.0)	NA	NA

Numbers and proportion vary because of missing data.

ASCUS: atypical squamous cells of undetermined significance; ASC-H: atypical squamous cells, cannot exclude high-grade squamous intraepithelial lesion (HSIL); AGC: atypical glandular cells; LSIL: low-grade squamous intraepithelial lesion; HSIL: high-grade squamous intraepithelial lesion; AIS: adenocarcinoma in situ. NA: Not available due to limited data;

Statically significant results are shown in bold.

1) Cervical cytology obtained within 270 days after latest invitation.

2) Cervical cytology obtained between 271 days to 3 years after latest invitation or 2.5 to 3 years after latest cervical cytology.

3) Cervical cytology obtained less than 2.5 years after latest cervical cytology.

4) Prevalence proportion difference (%) for “routine opportunistic testing” compared to “screened after invitation”.

5) Prevalence proportion ratio for “routine opportunistic testing” compared to “screened after invitation”.

6) Prevalence proportion difference (%) for “sporadic opportunistic testing” compared to “screened after invitation”.

7) Prevalence proportion ratio for “sporadic opportunistic testing” compared to “screened after invitation”.

8) Carcinoma including squamous and carcinoma adenocarcinoma.

9) Not suitable for diagnosis

10) Necrosis

**Table 3:** Cytological diagnosis for screened women stratified by age

	Screened after invitation^1^		Routine opportunistic testing^2^				Sporadic opportunistic testing^3^			
	n/N^4^	%	n/N^5^	%	PPD % (95 % CI)^6^	PPR (95 % CI)^7^	n/N^8^	%	PPD % (95 % CI)^9^	PPR (95 % CI)^10^
Cytological diagnosis										
Normal cells										
23-28	92,693/104,448	(88.7)	32,034/36,357	(88.1)	**-0.6 (-1.0;-0.2)**	0.99 (0.98;1.00)	9,141/10,417	(87.8)	**-0.6 (-1.6;-0.2)**	0.99 (0.98;1.00)
29-34	87,023/93,912	(92.7)	33,615/36,896	(91.1)	**-1.6 (-2.0;-1.2)**	0.98 (0.98;1.00)	11,887/13,004	(91.4)	**-1.3 (-1.8;-0.8)**	0.99 (0.98;1.00)
35-42	149,489/158,989	(94.0)	40,764/44,098	(92.4)	**-1.6 (-1.9;-1.3)**	**0.98 (0.98;0.99)**	16,960/18,280	(92.8)	**-1.2 (-1.6;-0.8)**	**0.99 (0.98;0.99)**
43-49	149,650/157,444	(95.0)	30,384/32,427	(93.7)	**-1.3 (-1.6;-1.0)**	**0.99 (0.98;0.99)**	15,878/16,889	(94.0)	**-1.0 (-1.4;-0.6)**	0.98 (0.99;1.00)
HSIL										
23-28	1,762/104,448	(1.7)	778/36,357	(2.1)	0.4 (-0.8;1.6)	**1.27 (1.17;1.38)**	156/10,417	(1.5)	-0.2 (-2.2;1.8)	0.89 (0.75;1.04)
29-34	1,217/93,912	(1.3)	631/36,896	(1.7)	0.4 (-0.8;1.6)	**1.26 (1.15;1.39)**	105/13,004	(0.8)	-0.5 (-0.2;1.3)	**0.59 (0.49;0.73)**
35-42	1,242/158,989	(0.8)	552/44,098	(1.3)	0.5 (-0.6;1.6)	**1.60 (1.45;1.77)**	89/18,280	(0.5)	-0.3 (-1.8;1.2)	**0.62 (0.50;0.77)**
43-49	570/157,444	(0.4)	267/32,427	(0.8)	0.4 (-0.8;1.6)	**2.27 (1.97;2.63)**	58/16,889	(0.3)	-0.1 (-1.6;1.4)	0.95 (0.72;1.24)
Carcinoma in situ + AIS										
23-28	22/104,448	(0.02)	8/36,357	(0.02)	0.0 (-1.4;1.4)	1.05 (0.47;2.35)	2/10,417	(0.02)	0.0 (-2.0;2.0)	0.91 (0.21;3.88)
29-34	30/93,912	(0.03)	19/36,896	(0.05)	0.02 (-1.2;1.2)	1.61 (0.91;2.86)	5/13,004	(0.04)	0.01 (-1.8;1.9)	1.20 (0.47;3.10)
35-42	37/158,989	(0.02)	15/44,098	(0.03)	0.01 (-0.9;0.9)	1.46 (0.80;2.66)	6/18,280	(0.03)	0.01 (-1.4;1.4)	1.41 (0.60;3.34)
43-49	17/157,444	(0.01)	3/32,427	(0.01)	0.0 (-1.2;1.2)	0.86 (0.25;2.92)	1/16,889	(0.01)	NA	0.55 (0.07;4.12)
Carcinoma^11^										
23-28	4/104,448	(0.00)	3/36,357	(0.01)	0.01 (-1.1;1.1)	2.16 (0.48;9.63)	1/10,417	(0.01)	NA	2.51 (0.28;22.4)
29-34	9/93,912	(0.01)	6/36,896	(0.02)	0.01 (-1.2;1.3)	1.70 (0.60;4.77)	0/13,004	(0.00)	NA	NA
35-42	17/158,989	(0.01)	20/44,098	(0.05)	0.04 (-1.0;1.1)	**4.18 (2.19;7.99)**	0/18,280	(0.00)	NA	NA
43-49	12/157,444	(0.01)	13/32,427	(0.04)	0.03 (-1.2;1.3)	**5.26 (2.40;11.5)**	1/16,889	(0.01)	NA	0.78 (0.10;5.97)

*HSIL*: high-grade squamous intraepithelial lesion, *AIS*: adenocarcinoma in situ, *NA*: Not available due to limited data

1) Cervical cytology obtained within 270 days after latest invitation.

2) Cervical cytology obtained between 271 days to 3 years after latest invitation or 2.5 to 3 years after latest cervical cytology.

3) Cervical cytology obtained less than 2.5 years after latest cervical cytology.

4) n: number of women with the cytological diagnosis within the age group, N:all women screened after invitation in the age group.

5) n: number of women with the cytological diagnosis within the age group, N:all women being routing opportunistic tested in the age group.

6) Prevalence proportion difference (%) for “routine opportunistic testing” compared to “screened after invitation”.

7) Prevalence proportion ratio for “routine opportunistic testing” compared to “screened after invitation”.

8) n: number of women with the cytological diagnosis within the age group, N:all women being sporadic opportunistic tested in the age group.

9) Prevalence proportion difference (%) for “sporadic opportunistic testing” compared to “screened after invitation”.

10) Prevalence proportion ratio for “sporadic opportunistic testing” compared to “screened after invitation”

11) Carcinoma including squamous and carcinoma adenocarcinoma.

Statically significant results are shown in bold

### Sensitivity analysis

In the sensitivity analysis, we used a cut-off value of ≤365 days (instead of ≤270 days) to differentiate between invited women and women having opportunistic testing and found that the proportion of invited women increased from 71.2 % to 76.0 %, while the proportion of women undergoing routine or sporadic opportunistic testing decreased from 20.7 % to 16.0 % and from 8.1 % to 8.0 %, respectively. Still, a higher proportion of HSIL was seen among women having routine opportunistic testing, compared to invited women (data not shown).

### Sociodemography and opportunistic testing

Unadjusted and adjusted associations between routine and sporadic opportunistic testing and sociodemographic factors are presented in Table 4. No multicollinearity was observed between any of the independent variables, with all VIF values ranging between 1.00 and 2.21.

**Table 4:** Associations between sociodemographic factors and undergoing routine or sporadic opportunistic testing

	Routine opportunistic testing^1^ vs. screened after invitation^3^		Sporadic opportunistic testing^2^ vs. screened after invitation^3^	
	Unadjusted	Adjusted	Unadjusted	Adjusted
	OR (95 % CI)	OR (95 % CI)	OR (95 % CI)	OR (95 % CI)
Age (years)				
23–28	1 (ref)	1 (ref)	1 (ref)	1 (ref)
29–34	**1.13 (1.11–1.15)**	1.02 (1.00–1.04)	**1.39 (1.35–1.43)**	**1.17 (1.13–1.20)**
35–42	**0.80 (0.78–0.81)**	**0.75 (0.73–0.76)**	**1.15 (1.12–1.18)**	**0.98 (0.95–1.00)**
43–49	**0.59 (0.58–0.60)**	**0.55 (0.54–0.56)**	**1.08 (1.05–1.10)**	**0.91 (0.89–0.94)**
Residential region				
Southern Denmark	1 (ref)	1 (ref)	1 (ref)	1 (ref)
North Denmark	**1.75 (1.71–1.79)**	**1.81 (1.77–1.85)**	**1.85 (1.79–1.91)**	**1.88 (1.82–1.95)**
Central Denmark	**1.25 (1.23–1.27)**	**1.25 (1.23–1.28)**	**1.20 (1.17–1.23)**	**1.21 (1.18–1.25)**
Capital Region of Denmark	**1.30 (1.28–1.32)**	**1.25 (1.22–1.27)**	**1.56 (1.52–1.60)**	**1.55 (1.51–1.59)**
Region Sealand	**1.07 (1.05–1.09)**	**1.10 (1.07–1.12)**	**0.81 (0.78–0.84)**	**0.80 (0.77–0.83)**
Ethnicity				
Danish	1 (ref)	1 (ref)	1 (ref)	1 (ref)
Western immigrants	**1.54 (1.49–1.58)**	**1.25 (1.21–1.30)**	**0.89 (0.85–0.93)**	1.00 (0.95–1.06)
Non-western immigrants	**1.19 (1.15–1.22)**	**1.08 (1.05–1.12)**	**0.89 (0.85–0.93)**	1.01 (0.96–1.07)
Marital status				
Single	**1.38 (1.36–1.40)**	**1.22 (1.20–1.23)**	**1.16 (1.13–1.19)**	**1.20 (1.17–1.23)**
Married/registered partnership	1 (ref)	1 (ref)	1 (ref)	1 (ref)
Cohabiting	**1.40 (1.38–1.42)**	**1.16 (1.14–1.18)**	**1.27 (1.25–1.30)**	**1.10 (1.08–1.13)**
Occupation				
Employed	1 (ref)	1 (ref)	1 (ref)	1 (ref)
Self-employed/chief executive	**1.06 (1.03–1.09)**	**1.15 (1.11–1.18)**	**1.15 (1.11–1.20)**	**1.14 (1.10–1.19)**
Unemployed/benefits^4^	**1.30 (1.28–1.33)**	**1.27 (1.25–1.30)**	**1.21 (1.18–1.25)**	**1.21 (1.17–1.25)**
Retired	NA	NA	NA	NA
Social welfare recipients	**1.89 (1.83–1.94)**	**1.68 (1.63–1.73)**	**1.30 (1.25–1.36)**	**1.31 (1.24–1.37)**
Other	**1.21 (1.18–1.24)**	**1.12 (1.09–1.15)**	**0.73 (0.70–0.76)**	**0.85 (0.82–0.89)**
Education (years)				
≤10	**1.25 (1.23–1.27)**	**1.12 (1.10–1.14)**	**1.10 (1.08–1.13)**	1.02 (1.00–1.05)
11–15	1 (ref)	1 (ref)	1 (ref)	1 (ref)
>15	**1.14 (1.13–1.16)**	**1.07 (1.05–1.08)**	**1.19 (1.17–1.21)**	**1.07 (1.05–1.10)**

Adjusted model: Adjusted for age groups, residential region, ethnicity, marital status, occupation, and education.

Statistically significant results are shown in bold.

*NA*: Not available due to limited data

1) Cervical cytology obtained between 271 days to 3 years after latest invitation or 2.5 to 3 years after latest cervical cytology.

2) Cervical cytology obtained less than 2.5 years after latest cervical cytology.

3) Cervical cytology obtained within 270 days after latest invitation.

4) State benefits in relation to sickness, education, leave benefits, disability pension, and student grants

Women who were age ≥35 years less often underwent routine opportunistic testing compared to the youngest women (23–28 years). Furthermore, women having routine opportunistic testing were more often western immigrants (adj. OR: 1.25, CI: 1.21–1.30), single (adj. OR: 1.22, CI: 1.20–1.23), or social welfare recipients (adj. OR: 1.68, CI: 1.63–1.73), or had either shorter (≤10 years) or longer (>15 years) education (adj. OR: 1.12, CI: 1.10–1.14 and OR: 1.07, CI: 1.05–1.08, respectively) than invited women. For women having sporadic opportunistic testing, we found the same tendencies except for ethnicity, for which no associations were found.

Compared with women in Southern Denmark, women in all other regions had elevated odds of undergoing routine opportunistic testing; however, this increase was most pronounced for women in North Denmark (adj. OR: 1.81, CI: 1.77–1.85). Also, for sporadic opportunistic testing, major differences between regions were found, with women living in North Denmark having the highest odds (adj. OR: 1.88, CI: 1.81–1.95) and those in Region Sealand having the lowest odds (adj. OR: 0.80, CI: 0.77–0.83) for having sporadic opportunistic testing compared to women living in Southern Denmark.

## Discussion

### Main findings

We found that 28.8 % of the cervical cytology was either the result of routine (20.7 %) or sporadic opportunistic testing (8.1 %). A larger proportion of women undergoing routine opportunistic testing were identified with HSIL abnormalities compared to invited women.Women tested in a shorter interval than recommended (sporadic opportunistic testing) had a similar risk of cytological abnormalities as invited women. Routine opportunistic testing was especially associated with being younger, a western immigrant, single, or a social welfare recipient. Similar associations were seen for undergoing sporadic opportunistic testing except for ethnicity, which was not associated with sporadic opportunistic testing. Residential region had the strongest associations with having either routine or sporadic opportunistic testing.

### Strengths and limitations

This study analyse for the first time the outcome of opportunistic testing in Denmark.

Linkage of Danish registries using the unique CRN made it possible to conduct a large-scale nationwide study that included data on cytological diagnosis and sociodemographic variables with high validity [21, 31]. The use of registry data meant that the study did not rely on self-reported data as an earlier study did [26], minimised selection and information bias, and enhanced the validity and generalisability of the study. A large sample and high statistical precision were ensured because this nationwide study included all women in the selected age group registered as having had a cervical cytology in the whole country within 3.5 years. Finally, because of coding history in the DPD, we could exclude very precisely those women who could be in a surveillance program and who therefore should not be in the study population.

A study limitation was the lack of information about the indication for cytology. Clinicians can obtain a cervical cytology in apparently healthy women, but recommendations also exist for taking a cervical cytology as part of the diagnostic procedure for relevant symptoms. We do not know the specific indications, and therefore the cytologies were categorised based on time since the last invitation or last cervical cytology. Opportunistic testing makes sense if a woman did not respond to her latest invitation or if she received a gynaecological examination just before an invitation was expected. Therefore, we chose to combine these two situations into one category “routine opportunistic testing”. Testing more often than the recommendations is more equivocal and cannot a priori be recommended. Therefore, these opportunistic cytologies were handled separately as “sporadic opportunistic cytologies”. The study population was limited to 23–49-year-old women. Further analyses will therefore be needed to evaluate if the results of this study also will apply for older women.

In addition, the use of the registries allowed inclusion in the study of various, almost complete variables. However, it is still possible that incorrect SNOMED coding has led to insufficient exclusion of women referred to a surveillance program; consequently, the proportion of opportunistic testing may be lower than estimated. Finally, in this study the cytological diagnoses were used as a proxy for the impact of screening as the histologically diagnoses were not available in our dataset.

### Comparison with other studies

In the British National Health Service Cervical Screening Program, Blanks and colleagues studied prevalence of opportunistic testing in the cervical screening program and estimated 72 % and 28 % of the primary cervical cytologies to be invitational and opportunistic, respectively [8]. However, the categorisation of samples differed between Blanks et al. [8] and our study, as Blanks et al. were able to identify if a cytology was initiated by an invitation (invitational testing) or initiated by the GP or the woman herself, while our definitions relied solely on the time since last cytology or last invitation. Furthermore, opportunistic testing was categorised differently in the two studies, thus the results cannot be directly compared.

Our study demonstrates that women undergoing routine opportunistic testing had a higher proportion of cytology abnormalities including HSIL than women who were screened after invitation. We likely obtained this clinically relevant result because the majority of women having routine opportunistic testing had a longer time interval since the latest cytology and therefore a priori would have had a higher risk of precancerous lesions. The results demonstrate the importance of catching up on individuals not following the recommended screening intervals to diagnose precancerous lesions in due time for preventive treatment.

Testing more often than the recommended screening interval (sporadic opportunistic testing) was examined in a UK study performed in clinics focused on sexually transmitted infections [13]. The authors found that sporadic opportunistic smears had marginally significantly increased rates of LSIL abnormalities but lower (not statistically significant) HSIL abnormalities than routine smears and concluded that there was no reason to depart from recommended screening intervals [13]. Their findings are in line with our results except that we identified no differences in cytological abnormalities between sporadic opportunistic cytologies and cytologies taken after invitation. In our study, this outcome is somewhat surprising because one reasonable expectation was that clinicians take cytology specimens at shorter screening intervals than recommended because of symptoms. It could be speculated that this result is because the group of women being sporadic opportunistic tested in our study is composed of those with a higher risk (symptomatic women) and those being screened more often than recommend and therefore having a lower risk of abnormalities. However, this cannot be further explored in our dataset.

In our study, younger age was associated with opportunistic testing, which is in line with earlier reports [8, 26, 32]. This association can probably be explained by the fact that younger women (especially ages 29–34) may be pregnant and therefore disrupt the regular scheduling of the systematic screening program.

Being an immigrant [17, 28], single [17, 33], or a social welfare recipient [34] or having an low education level [17] is associated with non-participation in cervical cancer screening programs. Our study identified these factors as associated with being opportunistically tested, suggesting that opportunistic testing may serve as a relevant alternative for some women who would not otherwise have been screened.

Residential region had the largest independent influence on opportunistic testing. A possible reason may be the unsystematic implementation of cervical cancer screening, which reached national coverage only in 2007. Regions without a systematic screening program offer had high opportunistic testing activity [6], and it may be that former routines regarding how and when to obtain a cervical cytology persisted in areas with the most recent implementation of the systematic screening program. It should be noted that participation-rates vary between 64 % and 70 % in the different regions which may also contribute to explain the regional differences in our study [18].

## Conclusion

This study categorised and analysed for the first time outcomes of opportunistic testing in Denmark and showed that one fourth of cervical cytologies were taken opportunistically. Women undergoing routine opportunistic testing had a higher proportion of cytological abnormalities and were more often underserved than women screened after invitation. Hence, routine opportunistic testing might serve as an important supplement to the systematic screening program by including non-participating women who may otherwise be tested with a delay or not tested at all. Benefits in terms of detecting more cytological abnormalities could not be identified for women tested more often than the recommended time interval (sporadically tested women).

## Appendix 1

**Table 5:** SNOMED codes

Modified World Health Organization classification	Normal cells	Atypical	Mild dysplasia	Moderate dysplasia	Severe dysplasia	Carcinoma in situ	Carcinoma	Inadequate
Bethesda classification	Normal cells	ASCUS, ASC-H, AGC	LSIL	HSIL			Carcinoma	Inadequate
SNOMED codes:	*M00100,M00120,M00121, M00122,M01111,M02561, M09450,M09462,M09463, M09460,M11600,M11610, M4…. AND NOT M09010, M51620, M58…, M69520, M69780 AND NOT M09010, M69784, M69810, M69820, M69880, M72…, M73… AND NOT M73005 OR M73225,OR M73229, OR M73309, M74030, MYY122*	ASCUS:*M67014, M69711, M72125, M73005, M69700, M73225*	*M67016, M74a.9, M69701,M76701, M76720,M69790 and not (M67014 OR M69711 OR M72125 OR M73005 OR M73225 OR M69700), M76700*	HSIL: *M67017, M69702, M69703, M69760, M740.9, M74c.9, M74b.9, M74HG9, M697X0.*			M8…3 OR M9…3, M80011, M80015	*M09010, M09011, M09012, M09013, M09014, M09015, M09016, M09017, M09018, M09019, M09070, M09000, M09100, M09140, M09145, M09150, M0901X, M0901Y, M30610, M37000, M69780, M54310*
				Carcinoma in situ: *M80702, M80732, M80762, M80812, M80102, M80722,*				
				Adenocarcinoma in situ: *M81402,M73309*				
		ASC-H:*M67010* AGC:*M67020, M69712, M69762*						
		ᅟ						
Women registered with a code with recommendation of control.								
SNOMED codes: ÆAA001, ÆAA002, ÆAA003, ÆAA004, ÆAA005, ÆAA006, ÆAA007, ÆAA008, ÆAA009, ÆAA00A, AA000B, ÆAA00E, ÆAA010, ÆAA011, ÆAA012, ÆAA013, ÆAA014, ÆAA015, ÆAA018, ÆAA019, ÆAA01G, ÆAA01H, ÆAA01I, ÆAA01K, ÆAA01Y, ÆAA020, ÆAA021, ÆAA0X0, ÆAA0X1, ÆAA0X4, ÆAA0X5, ÆAA0X7, ÆAA0Y0, ÆAA0Y1, ÆAA0Y2, ÆAA0Y3, ÆAA0Y4, ÆAAX15								
