# Erratum to: Impact of opportunistic testing in a systematic cervical cancer screening program: a nationwide registry study

**DOI:** 10.1186/s12889-015-2158-7

**Published:** 2015-09-14

**Authors:** Mette Tranberg, Mette Bach Larsen, Ellen M. Mikkelsen, Hans Svanholm, Berit Andersen

**Affiliations:** Department of Public Health Programs, Randers Regional Hospital, Skovlyvej 1, Randers, DK-8930 NØ Denmark; Department of Clinical Epidemiology, Aarhus University Hospital, Olof Palmes Allé 43-45, Aarhus N, DK-8200 Denmark; Department of Pathology, Randers Regional Hospital, Skovlyvej 1, Randers, DK-8930 NØ Denmark

Unfortunately, the original version of this article [1] contained an error. In the methods section page 2, lines 5–8 is written “Systematic cervical cancer screening was introduced in the 1960s in some counties and non-systematically implemented in the rest of the country until nationwide coverage was achieved in 2007 [19]”. This should be corrected to “Systematic cervical cancer screening was introduced in the 1960s in some counties and non-systematically implemented in the rest of the country until nationwide coverage was achieved in the late 1990s [7,19]”.

Furthermore, another error was detected in the discussion section under the headline “Comparison with other studies”. It is written: “A possible reason may the unsystematic implementation of cervical cancer screening, which reached national coverage only in 2007”. This should be corrected to “A possible reason may be the unsystematic implementation of cervical cancer screening, which only reached national coverage in the late 1990’s”.
